# Intimal cardiac sarcoma

**DOI:** 10.1093/ehjcr/ytae477

**Published:** 2024-09-03

**Authors:** Sylvain Diop, Vincent Thomas De Montpreville, Julien Guihaire

**Affiliations:** Department of Anesthesiology, Centre Chirurgical Marie Lannelongue, Groupe Hospitalier Paris Saint-Joseph, 92350 Le Plessis-Robinson, France; Department of Pathology, Centre Chirurgical Marie Lannelongue, Groupe Hospitalier Paris Saint-Joseph, Le Plessis-Robinson, France; Department of Cardiac Surgery, Centre Chirurgical Marie Lannelongue, Groupe Hospitalier Paris Saint-Joseph, Le Plessis-Robinson, France

**Figure:**
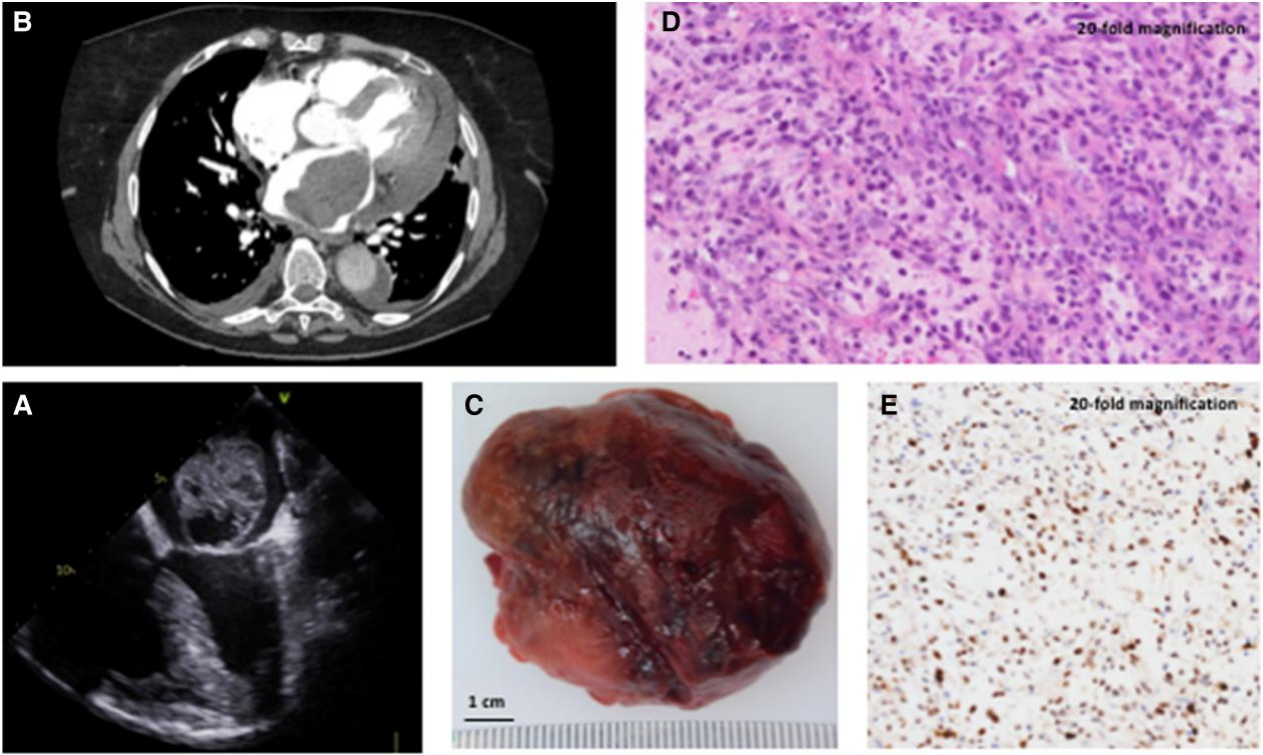


A 72-year-old woman without previous medical history was admitted to intensive care unit for acute onset of a New York Heart Association Class III dyspnoea with orthopnoea, heart palpitations, and crepitant rales consistent with the diagnosis of acute pulmonary oedema. Electrocardiogram showed atrial fibrillation at 130 b.p.m., and echocardiography revealed a large heterogeneous spherical mass into the left atrium protruding into the ventricle at each diastole and leading to the mechanical obstruction of the left ventricle filling (*Panel A*; see Supplementary material online, *Video S1*). Chest computed tomography scan imaging showed a 7 cm intra-atrial mass invading the left upper pulmonary vein, the pericardium, and the left pleura (*Panel B*). A diagnosis of an invasive malignant tumour was suspected. There was no evidence of distant metastases. A salvage open-heart surgery was decided to remove the intra-cardiac obstructive mass. Surgical examination under cardiopulmonary bypass revealed a solid tumour coming from the left upper pulmonary vein with an extravascular extension on the left atrial roof and on the left atrioventricular groove. The resection of the intra-cardiac mass was performed (*Panel C*). The proximal part of the tumour coming from the left upper pulmonary vein could not be removed due to major adhesions and extravascular invasion. Histologic analysis displayed typical spindle and pleomorphic cell sarcoma (*Panel D*; hematoxyline-eosine-safran coloration, 20-fold magnification). Immunohistochemical analysis showed an overexpression of murine double minute 2, which is a characteristic of intimal cardiac sarcoma (*Panel E*; brown nuclei, 20-fold magnification), an extremely rare type of aggressive sarcoma associated with very poor outcomes. Post-operative course was uneventful, and the patient was referred to an oncology specialist for initiation of adjuvant chemotherapy few weeks later.

## Supplementary Material

ytae477_Supplementary_Data

## Data Availability

Not applicable.

## Supplementary material

**Consent:** Written informed consent was obtained from the patient before submitting the manuscript.

**Funding:** None declared.

